# Supplementary material for: Genome wide genetic dissection of wheat quality and yield related traits and their relationship with grain shape and size traits in an elite × non-adapted bread wheat cross
Source: PLoS One. 2019 Sep 18;14(9):e0221826. doi: 10.1371/journal.pone.0221826 (PMC6750600; doi:10.1371/journal.pone.0221826)
Supplement: Attachment — Submitted filename: Response to reviewers comments.docx [file pone.0221826.s006.docx]

**Response to reviewer’s comments**

Journal Requirements:

1. When submitting your revision, we need you to address these additional requirements.

Please ensure that your manuscript meets PLOS ONE's style requirements, including those for file naming. The PLOS ONE style templates can be found at
http://www.journals.plos.org/plosone/s/file?id=wjVg/PLOSOne_formatting_sample_main_body.pdf and <http://www.journals.plos.org/plosone/s/file?id=ba62/PLOSOne_formatting_sample_title_authors_affiliations.pdf>

**Author response:** We have formatted the manuscript according to PLOS One style and hope that it meet the journal requirements.

2. We note that you have included the phrase “data not shown” in your manuscript. Unfortunately, this does not meet our data sharing requirements. PLOS does not permit references to inaccessible data. We require that authors provide all relevant data within the paper, Supporting Information files, or in an acceptable, public repository. Please add a citation to support this phrase or upload the data that corresponds with these findings to a stable repository (such as Figshare or Dryad) and provide and URLs, DOIs, or accession numbers that may be used to access these data. Or, if the data are not a core part of the research being presented in your study, we ask that you remove the phrase that refers to these data.

**Author response:** The phrase “data not shown” was removed from the manuscript.

[Note: HTML markup is below. Please do not edit.]

Reviewers' comments:

Reviewer's Responses to Questions

**Comments to the Author**

1. Is the manuscript technically sound, and do the data support the conclusions?

The manuscript must describe a technically sound piece of scientific research with data that supports the conclusions. Experiments must have been conducted rigorously, with appropriate controls, replication, and sample sizes. The conclusions must be drawn appropriately based on the data presented.

Reviewer #1: Yes

Reviewer #2: Yes

2. Has the statistical analysis been performed appropriately and rigorously?

Reviewer #1: Yes

Reviewer #2: Yes

3. Have the authors made all data underlying the findings in their manuscript fully available?

The [PLOS Data policy](http://www.plosone.org/static/policies.action#sharing) requires authors to make all data underlying the findings described in their manuscript fully available without restriction, with rare exception (please refer to the Data Availability Statement in the manuscript PDF file). The data should be provided as part of the manuscript or its supporting information, or deposited to a public repository. For example, in addition to summary statistics, the data points behind means, medians and variance measures should be available. If there are restrictions on publicly sharing data—e.g. participant privacy or use of data from a third party—those must be specified.

Reviewer #1: Yes

Reviewer #2: Yes

4. Is the manuscript presented in an intelligible fashion and written in standard English?

PLOS ONE does not copyedit accepted manuscripts, so the language in submitted articles must be clear, correct, and unambiguous. Any typographical or grammatical errors should be corrected at revision, so please note any specific errors here.

Reviewer #1: Yes

Reviewer #2: Yes

5. Review Comments to the Author

Please use the space provided to explain your answers to the questions above. You may also include additional comments for the author, including concerns about dual publication, research ethics, or publication ethics. (Please upload your review as an attachment if it exceeds 20,000 characters)

Reviewer #1: The following expresses my opinion about the merits of the study:

1) The paper provides useful information for breeders of hard red spring wheat and for the wider wheat community, particularly since the paper addresses the association of wheat quality and yield traits with grain shape and size traits, a poorly studied area.

2) The use of a high-density map for the mapping procedures should enable high efficiency application of MAS in future breeding programmes based upon this germplasm or upon these general results.

3) The identification of novel alleles provided by the non-adapted line, especially (i) alleles for improved GPC with no detrimental effects on yield or other traits, and (ii) alleles influencing grain hardness, together potentially provide new opportunities for achieving genetic progress in these traits.

4) Other valuable features of the paper are: (i) the detailed analysis of epistasis, which provides additional evidence of the importance of taking this into account during selection; (ii) the evidence presented that selection for grain area during breeding might also provide dividends; and (iii) the extensive comparison between the QTLs mapped here and those published previously, which details which of the QTLs identifies here are truly novel.

**Author response:** Thank you so much for your comments, wonderful observations about our manuscript and your recommendation for publication.

A comment: the study would probably have been more valuable still if more than 160 RILs had been evaluated and, to boot, more site-year combinations had been included; nonetheless, I do not see these drawbacks as sufficient motive to hold up publication of the study as it stands.

**Author response:** Thank you so much for your comment. We understand that larger sample size and more site-year combinations, may provide additional information. Larger population may help identify some additional minor QTL (if any). That being said, we strongly feel that (and as the results indicate), this study has identified important QTL with significant effects. In QTL mapping studies, it is a common and acceptable practice to have a population of >100 and minimum of three site-year combinations (our study used data for 160 RILs from four site-year combinations). One of the main reason for this is the large amount of resources required to carry out field evaluation at multiple locations. Moreover, the phenotypic assays for evaluation of quality traits are labor intensive and require huge amount of resources, thus limiting site-year combinations to a manageable and acceptable number.

Am I right in thinking that there is disagreement in the labelling of the parental phenotypic mean values between Table 1 and Fig. 1? I recommend checking this.

**Author response:** Thank you so much for noticing this mistake. We have now made necessary corrections to the legend of Figure 1.

Grammatically the writing is generally clear, although it might be further improved by being looked over once more.

**Author response:** Thank you for your comment. As can be seen in the manuscript, we have gone through the manuscript one more time to improve the manuscript.

Based upon the above opinion, I feel the paper is well worthy of publication.

Reviewer #2: The manuscript has some new finding in the area (specified for hard red spring wheat) which can be useful in the breeding program.
It has dissected the genetics of wheat quality and yield traits and also identified novel QTL and markers closely associated with quality and yield. It shows data about the epistasis in the genetic control of quality and yield as well.
The manuscript is well written. My only concern is that only two replicates were used in this experiment. I think it should be more.
I think it is suitable for publication.

**Author response:** Thank you so much for your review of our manuscript and your recommendation.

We understand that more replications are always better, but due to resource and labor intensive phenotyping in multi-environment mapping studies, it is a standard and acceptable practice to have two replicates per environment. Moreover, the phenotypic assays for evaluation of quality traits are highly labor intensive and require huge amount of resources, thus limiting number of replicates and site-year combinations to a manageable and acceptable number.
