# Supplementary material for: Genome wide genetic dissection of wheat quality and yield related traits and their relationship with grain shape and size traits in an elite × non-adapted bread wheat cross
Source: PLoS One. 2019 Sep 18;14(9):e0221826. doi: 10.1371/journal.pone.0221826 (PMC6750600; doi:10.1371/journal.pone.0221826)
Supplement: Attachment — Submitted filename: Response to reviewers comments.docx [file pone.0221826.s007.docx]

Reviewer #1: As requested, the authors have (i) adequately defended the questions posed on sample size and the number of site-year combinations included; (ii) corrected the discrepancy between Table 1 and Fig. 1; and (iii) improved the grammar.

I therefore maintain my original opinion that the paper is well worthy of publication.

Nonetheless, below I include additional modifications to the grammar, those that I have managed to identify in the time available:

Personally, I would generally replace “QTL” with “QTLs” where it is used in the plural, but do not insist since papers vary in this respect.

The following are more specific modifications suggested to particular lines in the manuscript:

l. 56: insert “the” after “while”.

l. 59: insert “it” after “although”.

l. 60: replace “they do” with “does”.

l. 61: insert “with them” after “loci”, insert “A” before “major”, insert “the” before “Rht8” and insert “the” before “reduced”.

l. 62: replace “increase” with “increases”.

l. 97: replace “more” with “a greater”.

l. 259: insert “The” before “same”.

l. 277: replace “negatively” with “negative”.

l. 298: replace “has” with “had”.

l. 299: replace “effect” with “effects”.

l. 314: replace the phrase with “The majority (52 out of 60) of these QTL”.

l. 362: insert “the” before “PI”.

l. 363: insert “the” before “PI”.

l. 364: insert “the” before “ND”.

l. 415: replace “,” with “and” and insert “,” after “SPMS”.

l. 420: replace “had association” with “were associated”.

l. 432: replace “those” with “these”.

l. 434: insert “the” before “PI” and replace “were” with “was”.

l. 440: replace “interaction” with “interactions”.

l. 444: replace “More” with “A greater”.

l. 455: delete “each” and insert “for each trait” after “detected”.

l. 456: replace “locus” with “loci”.

l. 457: replace “interaction” with “interactions”.

l. 458: delete “or”.

l. 473: replace “helps” with “help” and replace “marker” with “market”.

l. 484: insert “a” after “of”.

l. 491: insert “,” after “effect”.

l. 494: insert “the” after “that”, replace “increase” with “increased” and delete “Like this study”.

l. 495: delete “as well”.

l. 500: insert “the” after “by” and insert “,” after “genotype”.

l. 502: insert “the” before “contribution” and “the” before “majority”.

l. 505: insert “,” after “yield”.

l. 506: insert “,” after “traits”.

l. 508: delete “,”.

l. 510: delete “,” after “Although”.

l. 513: delete “,” before “were”.

l. 515: replace “have” with “has”.

l. 516: insert “,” after “however”.

l. 519: replace “difference” with “differences”.

l. 520: insert “,” before “does”.

l. 521: insert “the” after “as”.

l. 522: insert “the” after “allele of”.

l. 523: insert “a” before “functional”.

l. 525: insert “the” before “high” and insert “the” before “Gpc-B1”.

l. 529: insert “,” before “where”.

l. 531/2: delete “which is”.

l. 534: insert “,” after “proteins”.

l. 537: insert “the” after “of”.

l. 538: delete “as well”.

l. 539: delete “,” after “genotypes”.

l. 541: replace “major” with “greater”.

l. 553: delete “,” after “(SPMS)”.

l. 554: insert “the” after “three”.

l. 556: replace “trait” with “traits”, insert “,” after “traits” and delete “which is”.

l. 560: insert “,” before “is”.

l. 564: replace “effect” with “effects”.

l. 566: replace “effect” with “effects”.

l. 568: insert “,” after “alleles”, replace “increase” with “they increased” and delete “they”.

l. 569: replace “decrease” with “decreases” and insert “,” after “components”.

l. 570: replace “environment” with “environments”.

l. 575: replace “an” with “on”.

l. 583: replace “Till” with “To”.

l. 585: insert “the” after “to”.

l. 586: insert “the” after “in”.

l. 587: insert “,” after “2D”.

l. 588: replace “papulation” with “population”.

l. 589: insert “the” before “alleles”.

l. 599: insert “the” after “since”.

l. 601: insert “the” before “Japonese”.

l. 604: delete “,” after “although” and replace “differ” with “differed”.

l. 607: insert “the” before “Rht8” and insert “a” before “larger”.

l. 608: replace “suggest” with “suggests”.

l. 609: insert “the” after “at”.

l. 617: insert “the” after “at”.

l. 618: insert “the” after “at”.

l. 619: delete “,”, insert “a” after “showed” and insert “the” after “in”.

l. 620: insert “the” after “of”.

l. 623: insert “,” after “estimates”.

l. 626: insert “the” after “while”.

l. 627: insert “the” after “by” and insert “the” after “using”.

l. 628: delete “,” after “[86]”.

l. 629: delete “,” after “Since”.

l. 630: insert “the” after “harbors” and replace “alleles” with “allele”.

l. 632: insert “the” after “either” and replace “loci” with “locus”.

l. 634: delete “,”.

l. 635: insert “the” after “of” and replace “gene” with “genes”.

l. 641: insert “,” after “is”, insert “,” after “important” and delete “but”.

l. 643: delete “although” and insert “although” after “,”.

l. 647: delete “,” after “Although”.

l. 655: replace “more” with “a greater”.

l. 657: delete “,”.

l. 659: delete “,” after “thus”.

l. 660: replace “component” with “components” and delete “,” after “Since”.

l. 661: delete “,”.

l. 667: insert “,” after “crucial”.

l. 668: delete “,” and replace “effects” with “affects”.

l. 672: insert “,” after “own”.

l. 674: replace “has” with “have”.

l. 675: insert “The” before “Role” (role).

l. 677: insert “a” after “contributed”.

l. 681: delete “,”.

l. 692: replace “trait” with “traits”.

l. 698: delete “,”.

l. 700: insert “,” after “GA”.

l. 704: insert “,” after “trait” and delete “an”.

l. 707: insert “,” before “which” and insert “the” before “allele”.

l. 710: delete “a”.

l. 713: insert “be” after “to” and delete “the”.

l. 714: replace “program” with “programs”.

l. 715: delete “~”.

l. 721: delete “an”.

**Authors’ response:** We HIGHLY appreciate the reviewer for thoroughly going through our manuscript and for suggesting all the corrections. We have gone through the whole manuscript again and made all the necessary corrections in the grammar (using track changes).
